# Clinical Profile and Association Between Admission Glasgow Coma Scale and In-Hospital Mortality Among Children With Head Injury at a Tertiary Care Centre in Central India

**DOI:** 10.7759/cureus.114780

**Published:** 2026-08-19

**Authors:** Saket Jha, Samriddhi Jain, Abhilash Tiwari, Utkarsh Bhandari, Deepak K Nayak, Gaurav Gupta

**Affiliations:** 1 Pediatric Surgery, Gandhi Medical College, Bhopal, IND; 2 Neonatology, Rainbow Children's Hospital, Delhi, IND; 3 Pediatric Medicine, Gandhi Medical College, Bhopal, IND; 4 Urology, Max Super Speciality Hospital, Noida, IND; 5 Urology, Gandhi Medical College, Bhopal, IND; 6 Neurosurgery, Apollo Hospitals, Bhubaneswar, IND; 7 Neurosurgery, Netaji Subhash Chandra Bose Medical College, Jabalpur, IND

**Keywords:** central india region, fall from height, glasgow coma scale (gcs), in‑hospital mortality, pediatric head injury, retrospective study, traumatic brain injury (tbi)

## Abstract

Introduction

Traumatic brain injury (TBI) is a leading cause of trauma-related morbidity and mortality in children worldwide, and injury patterns in low- and middle-income countries differ from those reported in high-income settings. Data from tertiary centres in central India remain limited. This study describes the clinical profile, management pattern, and in-hospital outcome of children admitted with head injury at a tertiary care centre and evaluates the association between admission Glasgow Coma Scale (GCS) and in-hospital mortality.

Methods

This retrospective observational study included 81 children admitted with head injury to the Department of Pediatric Surgery, Gandhi Medical College, Bhopal, India, between January 2026 and June 2026. Demographic details, mechanism of injury, clinical features at presentation, admission GCS, non-contrast computed tomography (NCCT) head findings, management type, length of hospital stay, and outcome were recorded from case records. The association between GCS severity and outcome was assessed using Fisher's exact test, the chi-square test, and the Mann-Whitney U test, with p < 0.05 considered significant.

Results

The mean age was 5.03 ± 3.31 years, with a male-to-female ratio of 1.53:1. Fall from height was the commonest mechanism (44 of 81, 54.3%), followed by road traffic accidents (17 of 81, 21.0%). Most children presented with mild head injury (GCS 13-15; 61 of 81, 75.3%); eight (9.9%) had severe injury (GCS ≤8). NCCT was performed in 78 of 81 children (96.3%); skull fracture (37 of 78, 47.4%) was the most common finding, followed by extradural hemorrhage (20 of 78, 25.6%) and subdural hemorrhage (18 of 78, 23.1%). Conservative management was employed in 79 of 81 children (97.5%), with two (2.5%) requiring operative intervention. Overall in-hospital mortality was 11.1% (9 of 81). On Fisher's exact test, children with mild GCS had significantly lower odds of death compared with the combined moderate-to-severe group used as the reference category (odds ratio, 0.03; 95% CI, 0.003-0.22; p = 0.00003); seven of eight children with severe GCS died, compared with one of 61 with mild GCS. Mean GCS was significantly lower among children who died than among those discharged (6.1 versus 14.0; Mann-Whitney U = 23.0; p = 0.00000024).

Conclusions

Pediatric head injury at this centre was predominantly mild and managed conservatively, with fall from height as the leading mechanism, consistent with other Indian series. Lower admission GCS was strongly associated with in-hospital mortality on unadjusted analysis. These findings are descriptive and hypothesis-generating within this cohort; larger, prospective, multicentric studies with multivariable analysis are needed to confirm this association and identify additional factors influencing outcome.

## Introduction

Traumatic brain injury (TBI) refers to an alteration in brain function or other evidence of brain pathology caused by an external force, such as a blow, jolt, or penetrating injury to the head. Its clinical presentation spans a wide spectrum, ranging from transient confusion or brief loss of consciousness in mild cases to prolonged unconsciousness, focal neurological deficits, or death in more severe cases, depending on the mechanism, force, and location of injury. Severity is most commonly assessed at the bedside using the Glasgow Coma Scale (GCS), a simple, standardized tool that scores eye-opening, verbal, and motor responses to grade the level of consciousness and guide early clinical decision-making. In children, the leading causes of head injury include falls, road traffic accidents, and, less commonly, sports-related or inflicted injuries, with the eventual clinical course shaped by both the severity of the initial injury and the timeliness of subsequent medical care.

TBI is one of the leading causes of death and disability among children worldwide and constitutes a major, preventable public health burden [1]. Global estimates place the incidence of pediatric TBI at over 200 per 100,000 children annually, with the burden disproportionately affecting low- and middle-income countries, where trauma systems, pre-hospital care, and injury-prevention infrastructure remain limited [1]. In India, head injury is reported to account for a substantial proportion of pediatric trauma presentations, with falls and road traffic accidents consistently identified as the two dominant mechanisms across multiple tertiary-centre series [2,3].

Pediatric head injury presents distinct assessment challenges compared with adult head injury, as tools such as the GCS were originally developed and validated in adults and require age-specific adaptation for reliable use in young and pre-verbal children. Despite this, the GCS remains the most widely used bedside tool for the initial assessment and severity stratification of pediatric head injury, and admission GCS has repeatedly been shown to correlate with short-term outcome in Indian and other regional cohorts [4,5].

Central India, including the state of Madhya Pradesh, has a large rural population with limited access to organized trauma care and pre-hospital transport, which may influence both the pattern of injury and the timeliness of presentation to tertiary centres. However, published data describing the clinical profile and outcome of pediatric head injury from this region remain sparse, and no prior study from a government tertiary care centre in central India has characterized this population. This study does not aim to establish a novel association between GCS and mortality, which is already well documented in the literature; rather, it aims to provide descriptive regional data on the demographic and clinical profile, management pattern, and outcome of children admitted with head injury at a tertiary care centre in Bhopal and to confirm whether the established GCS-mortality association holds in this previously uncharacterized population and setting.

## Materials and methods

This was a retrospective observational study conducted in the Department of Pediatric Surgery, Gandhi Medical College, Bhopal, a government-funded tertiary care teaching hospital in central India serving both rural and urban populations. This department is the sole service admitting pediatric head injury cases at the institution; children are not independently admitted under Neurosurgery, Pediatric Intensive Care, or General Pediatrics, so this cohort reflects all pediatric head injury admissions at the centre during the study period. The study followed exemption from full review by the Institutional Ethics Committee, Gandhi Medical College, Bhopal (IEC Protocol No. 15/IEC/2026; Letter No. 32314/MC/IEC/2026, dated July 15, 2026; Ethics Committee Registration No. ECR/1055/Inst/MP/2018). Although the study period (January-June 2026) reflects routine clinical care, record access and analysis began only after this exemption was granted. Patient data were de-identified by replacing names with sequential case identification numbers prior to analysis.

Children were included if they presented with a history of head trauma warranting hospital admission, irrespective of GCS, imaging findings, or clinical signs; confirmed brain injury on imaging was not required. Eligibility reflected the department's standing admission scope (children under 14 years) rather than a study-specific criterion; children discharged directly from the emergency department without admission were not included. Of 110 children screened during the study period, 29 (26.4%) were excluded for incomplete records (missing one or more of the following: age, sex, mechanism of injury, admission GCS, management type, or outcome), leaving 81 children (73.6%) for analysis; a reason-specific breakdown was not systematically logged. The final cohort's observed age range was 3 months to 13 years, with no neonates.

Data extracted included age, sex, place of injury (rural or urban, based on the documented injury location), mechanism of injury, clinical features at presentation (loss of consciousness, vomiting, bleeding from the ear/nose/throat, and seizure), admission GCS, non-contrast computed tomography (NCCT) findings, management type, hospital stay, and outcome. Admission GCS was the score recorded in our emergency department on arrival, prior to any resuscitation, sedation, or paralytic agents (no child in this cohort was sedated or paralyzed before assessment); for referred children, this reflects GCS at arrival at our institution, not any pre-transfer score. Standard adult GCS was applied uniformly, including in pre-verbal children, rather than the pediatric/modified GCS, reflecting actual clinical practice. Where GCS was recorded in component form, the total was derived by summation; intubated children (n = 2) were assigned the lowest possible verbal score in the absence of a documented pre-intubation value. GCS was categorized as mild (13-15), moderate (9-12), or severe (8 or less).

Mechanism of injury was categorized as fall from height (fall from an elevated surface such as a roof, terrace, tree, or window), fall from stairs, slip and fall (same-level fall without elevation), and other mutually exclusive categories as documented in case records. NCCT findings (extradural hemorrhage, subdural hemorrhage, subarachnoid hemorrhage, hemorrhagic contusion, diffuse axonal injury, skull fracture, and pneumocephalus) were not mandatory for inclusion; three children did not undergo imaging. Diffuse axonal injury was suspected on NCCT and clinical grounds only, as MRI was not performed in this cohort. Conservative management was defined as non-operative management with observation, serial neurological assessment, and supportive care, without surgical evacuation.

Descriptive statistics were reported as mean and standard deviation or median and interquartile range for continuous variables, and frequency with percentage for categorical variables. Outcome was coded as death versus discharge (reference category). The primary analysis was Fisher's exact test comparing mild GCS against the combined moderate-to-severe group as the reference category, reflecting a clinically actionable threshold. As supplementary analyses, a chi-square test assessed the association across all three GCS strata, and the Mann-Whitney U test compared raw GCS scores and hospital stay between outcome groups. A sensitivity analysis repeated the primary comparison excluding intubated children. A p-value of less than 0.05 was considered significant. Analysis was performed using Python (version 3) with pandas and SciPy.

## Results

A total of 81 children admitted with head injury were included in the study, out of 110 children screened during the study period. The mean age was 5.03 ± 3.31 years (median, 4.0 years; range, 0.25-13 years), and the majority (55 of 81, 67.9%) belonged to the 3-to-11-year age group. There were 49 boys and 32 girls (49 of 81, 60.5%, and 32 of 81, 39.5%, respectively), giving a male-to-female ratio of 1.53:1. The place of injury was almost equally distributed between rural (41 of 81, 50.6%) and urban (40 of 81, 49.4%) areas.

Fall from height was the most common mechanism of injury, accounting for 44 of 81 children (54.3%), followed by road traffic accidents (17 of 81, 21.0%) and falls from stairs (8 of 81, 9.9%). Less common mechanisms included animal-related injury (being hit by a cow; 4 of 81, 4.9%), slip and fall (4 of 81, 4.9%), wall collapse (2 of 81, 2.5%), and single cases each of gunshot injury and assault (1 of 81, 1.2% each).

On clinical assessment at presentation, a history of loss of consciousness was noted in 39 of 81 children (48.1%), vomiting in 40 of 81 (49.4%), bleeding from the ear, nose, or throat in 44 of 81 (54.3%), and seizure in 8 of 81 (9.9%). Based on admission GCS, 61 of 81 children (75.3%) had mild head injury, 12 of 81 (14.8%) had moderate injury, and 8 of 81 (9.9%) had severe injury. The mean admission GCS for the entire cohort was 13.09 ± 3.31.

NCCT was performed in 78 of 81 children (96.3%); three children did not undergo imaging, all of whom were among the nine in-hospital deaths, most likely reflecting clinical instability that precluded transport for imaging. NCCT findings, with corresponding counts, are categorized in Table 1. Skull fracture was the most frequent finding (37 of 78, 47.4%), followed by extradural hemorrhage (20 of 78, 25.6%), subdural hemorrhage (18 of 78, 23.1%), subarachnoid hemorrhage (14 of 78, 17.9%), and hemorrhagic contusion (13 of 78, 16.7%). Pneumocephalus was noted in 6 of 78 children (7.7%), and suspected diffuse axonal injury, based on NCCT and clinical findings rather than MRI confirmation, was noted in 2 of 78 (2.6%). NCCT was reported as showing no abnormality in 2 of 78 children (2.6%).

**Table 1:** Demographic and clinical characteristics of children with head injury (N = 81) *NCCT was performed in 78 of 81 children (96.3%); percentages for NCCT findings are calculated using 78 as the denominator. Individual patients could have more than one finding, and percentages therefore do not sum to 100%. **Suspected diffuse axonal injury based on NCCT findings and clinical picture; not MRI-confirmed, as MRI was not performed in these cases. NCCT: non-contrast computed tomography; MRI: magnetic resonance imaging

Variable	Category	n (%)
Age group	Infant (<1 year)	7 (8.6)
	Toddler (1-2 years)	15 (18.5)
	Child (3-11 years)	55 (67.9)
	Adolescent (12+ years)	4 (4.9)
Sex	Male	49 (60.5)
	Female	32 (39.5)
Place of injury	Rural	41 (50.6)
	Urban	40 (49.4)
Mechanism of injury	Fall from height	44 (54.3)
	Road traffic accident	17 (21.0)
	Fall from stairs	8 (9.9)
	Animal-related (hit by cow)	4 (4.9)
	Slip and fall	4 (4.9)
	Wall collapse	2 (2.5)
	Gunshot injury	1 (1.2)
	Assault	1 (1.2)
GCS severity at admission	Mild (13-15)	61 (75.3)
	Moderate (9-12)	12 (14.8)
	Severe (8 or less)	8 (9.9)
Clinical features	History of loss of consciousness	39 (48.1)
	Vomiting	40 (49.4)
	Bleeding from ear, nose or throat	44 (54.3)
	Seizure	8 (9.9)
NCCT findings* (n = 78 scanned)	Skull fracture	37 (47.4)
	Extradural hemorrhage	20 (25.6)
	Subdural hemorrhage	18 (23.1)
	Subarachnoid hemorrhage	14 (17.9)
	Hemorrhagic contusion	13 (16.7)
	Pneumocephalus	6 (7.7)
	No abnormality detected	2 (2.6)
	Suspected diffuse axonal injury**	2 (2.6)
Management	Conservative	79 (97.5)
	Operative	2 (2.5)
Outcome	Discharged	72 (88.9)
	Death	9 (11.1)

The median duration of hospital stay was 4.0 days (interquartile range, 3.0-6.0 days; range, 1-28 days). The vast majority of children (97.5%, 79 of 81) were managed conservatively; only two children (2.5%) underwent operative intervention, both for evacuation of extradural hemorrhage.

Overall, 72 of 81 children (88.9%) were discharged, and nine (11.1%) died, giving an in-hospital case fatality rate of 11.1%. Admission GCS severity showed a strong association with outcome (Table 2). Among children with severe GCS (8 or less), seven of eight (87.5%) died, compared with one of 12 (8.3%) with moderate GCS and one of 61 (1.6%) with mild GCS. On Fisher's exact test, children with mild GCS had significantly lower odds of death compared with the combined moderate-to-severe group used as the reference category (odds ratio, 0.03; 95% CI, 0.003-0.22; p = 0.00003). A chi-square test across all three GCS severity strata confirmed this association (chi-square = 52.90; degrees of freedom = 2; p < 0.00001). Mean admission GCS was significantly lower among children who died (6.1 ± 3.4) than among those discharged (14.0 ± 1.6; Mann-Whitney U = 23.0; p = 0.00000024). Duration of hospital stay did not differ significantly between children who died (median, 2.0 days) and those discharged (median, 4.0 days; Mann-Whitney U = 197.0; p = 0.054), reflecting early mortality among the most severely injured children. Two children were intubated at presentation and assigned the lowest possible verbal score in the absence of a documented pre-intubation GCS. As a sensitivity analysis excluding these two children (n = 79), the association between GCS severity and mortality remained materially unchanged (odds ratio, 0.033; 95% CI, 0.004-0.303; p = 0.0004).

**Table 2:** Association between admission Glasgow Coma Scale severity and outcome Percentages are row-wise (percentage of each GCS severity group with the given outcome). Fisher's exact test (mild versus moderate-to-severe), odds ratio 0.03, 95% CI 0.003-0.22, p = 0.00003. Chi-square test across all three severity strata, chi-square = 52.90, degrees of freedom = 2, p < 0.00001.

GCS severity at admission	Discharged, n (%)	Death, n (%)	Total, n
Mild (13-15)	60 (98.4)	1 (1.6)	61
Moderate (9-12)	11 (91.7)	1 (8.3)	12
Severe (8 or less)	1 (12.5)	7 (87.5)	8
Total	72 (88.9)	9 (11.1)	81

Both operatively managed children had extradural hemorrhage; one of these children, injured by assault and with a severe admission GCS, died despite surgical evacuation, while the other, injured in a road traffic accident and with a moderate GCS, was discharged. Of the nine deaths, the mechanism of injury was fall from height in four, road traffic accident in two, and wall collapse, gunshot injury, and assault in one each.

## Discussion

This cohort's demographic and injury profile - mean age 5.03 years, male predominance (1.53:1), and fall from height as the leading mechanism (54.3%) - closely parallels a recent prospective North Indian cohort of 244 children (mean age 5.04 years, similar male preponderance, falls in 46.3%) [2], and is consistent with fall from height as the dominant mechanism of pediatric head injury across Indian series generally [2,3,6]. This concordance across geographically distinct centres suggests a shared, modifiable injury pattern - young children falling from unsecured heights - that may warrant targeted regional prevention measures such as window guards and balcony railings, rather than reflecting a phenomenon specific to this centre alone.

The near-equal rural-urban distribution observed in this cohort (50.6% versus 49.4%) is consistent with the substantial rural population of central India that this centre serves. We did not measure catchment area boundaries or pre-hospital transport times in this study, and this distribution should be interpreted descriptively rather than as evidence of specific referral patterns or transport delays; we raise this only as a plausible area for future study rather than a finding of the present analysis.

The proportion of children with mild head injury in this series (75.3%) is broadly similar in magnitude to figures reported from a large apex trauma centre in North India (85% among all emergency department evaluations; 64% among hospitalized inpatients specifically) [4], though these percentages are derived from different source populations and denominators (all-comers versus admitted versus inpatient subgroups) and are not directly comparable. Differences in referral patterns or case-mix may plausibly contribute to this variation, though neither factor was measured in either study, and this explanation should be regarded as speculative rather than established.

The predominant use of conservative management in this cohort (97.5%) is consistent with the observation across pediatric TBI literature that the majority of children, even those with structural findings such as skull fracture or small extra-axial hemorrhage, can be managed non-operatively with close clinical and radiological monitoring [2,4]. The decision to manage extradural and subdural hemorrhage conservatively versus operatively was based on lesion volume, degree of midline shift, and GCS trajectory rather than lesion type alone; the majority of hemorrhages in this cohort were small-volume without significant mass effect. Only two children in this series required operative intervention, both for extradural hemorrhage. Of the three children with extradural or subdural hemorrhage who died without surgical intervention, all presented with severe admission GCS and survived only one to seven days, suggesting these children were too critically unstable at presentation for surgery to be a feasible option, rather than being triaged away from surgery based on lesion characteristics. The fatal outcome in one of the two operative cases, despite surgical evacuation, further underscores that surgery does not guarantee survival when injury severity is profound at presentation.

The overall mortality of 11.1% in this cohort is higher than the 6.14% reported in a recent prospective North Indian series [2] and the 3% reported by Madaan et al. [4], but comparable to mortality rates reported from other resource-limited settings in the region [7]. The reason for this difference was not examined in this study and cannot be determined from the available data. Possible untested contributors that could be explored in future prospective work include differences in case-mix, referral patterns, and pre-hospital delay, none of which were measured here.

The central finding of this study is the strong association between admission GCS and mortality, with seven of eight children with severe GCS at presentation succumbing to their injury, compared with only one of 61 children with mild GCS. This finding is consistent with evidence that lower GCS, and particularly the motor component, is strongly and consistently associated with inpatient mortality in Indian pediatric and adult TBI cohorts [5,8]. This finding is consistent with the well-established role of GCS in head injury assessment generally; however, we did not evaluate its performance as a triage or counselling tool in this study, and any such application would require prospective validation with adjustment for confounders.

This study has several limitations. It is a retrospective, single-centre study restricted to children admitted under the Department of Pediatric Surgery; children with minor head injury discharged directly from the emergency department without admission are not represented, and cause of death was recorded as all-cause in-hospital mortality rather than mortality specifically attributed to head injury. Of 110 children screened, 29 (26.4%) were excluded for incomplete records, and a detailed reason-specific breakdown was not available; we cannot exclude the possibility of complete-case selection bias. With only nine deaths, this study was not powered a priori, and several subgroup estimates, particularly within Table 2, carry wide confidence intervals and should be interpreted as exploratory. Multivariable analysis to adjust for potential confounders such as age, mechanism of injury, and NCCT findings was not performed, as the small number of deaths would have resulted in fewer than 10 events per predictor variable; GCS was therefore evaluated as a single, unadjusted variable, and residual confounding cannot be excluded. Standard adult GCS was applied uniformly across the cohort, including infants and pre-verbal toddlers, rather than the pediatric/modified GCS, which may have overestimated injury severity in younger children. Similarly, a reliable pre-intubation GCS was not available for the two intubated children in this cohort, though a sensitivity analysis excluding these children showed that the primary association was materially unchanged. This study also did not capture quantitative lesion size, midline shift, serial neurological status, intensive care-level interventions, or a structured surgical eligibility assessment; the high rate of conservative management (97.5%) should therefore be interpreted as a descriptive summary of management patterns rather than evidence of its clinical appropriateness in each individual case. Cases labeled as diffuse axonal injury were based on NCCT and clinical findings rather than MRI confirmation. Finally, outcome was assessed only at discharge, and longer-term neurological and functional outcomes were not captured; larger, prospective, multicentric studies with structured follow-up and multivariable analysis are needed to validate and extend these findings.

## Conclusions

In this retrospective single-centre cohort, pediatric head injury was predominantly seen in young children following falls from height, was managed conservatively in the large majority of cases, and carried an overall in-hospital mortality of 11.1%. Lower admission GCS was strongly associated with in-hospital mortality on unadjusted analysis. These findings should be interpreted as descriptive and hypothesis-generating within this cohort, given the retrospective design, small number of deaths, and absence of confounder adjustment; larger, prospective, multicentric studies with multivariable analysis are needed before this association can inform clinical triage, counseling, or generalization to other settings.
